# A transgene design for enhancing oil content in Arabidopsis and Camelina seeds

**DOI:** 10.1186/s13068-018-1049-4

**Published:** 2018-02-21

**Authors:** Yerong Zhu, Linan Xie, Grace Q. Chen, Mi Yeon Lee, Dominique Loque, Henrik Vibe Scheller

**Affiliations:** 1 Joint BioEnergy Institute and Environmental Genomics and Systems Biology Division, Lawrence Berkeley National Laboratory, Berkeley, CA 94720 USA; 2 College of Life Science, Nankai University, Tianjin, 300071 China; 3 College of Life Science, Northeast Forestry University, Harbin, 150040 China; 4 Western Regional Research Center, Agricultural Research Service, U.S. Department of Agriculture, 800 Buchanan Street, Albany, CA 94710 USA; 5 Department of Plant and Microbial Biology, University of California Berkeley, Berkeley, CA 94720 USA

**Keywords:** *ZmLEC1*, Master regulator, Transcription factor, Oil content, SCPL17, ACP5

## Abstract

**Background:**

Increasing the oil yield is a major objective for oilseed crop improvement. Oil biosynthesis and accumulation are influenced by multiple genes involved in embryo and seed development. The *leafy cotyledon1* (*LEC1*) is a master regulator of embryo development that also enhances the expression of genes involved in fatty acid biosynthesis. We speculated that seed oil could be increased by targeted overexpression of a master regulating transcription factor for oil biosynthesis, using a downstream promoter for a gene in the oil biosynthesis pathway. To verify the effect of such a combination on seed oil content, we made constructs with maize (*Zea mays*) *ZmLEC1* driven by serine carboxypeptidase-like (*SCPL17*) and acyl carrier protein (*ACP5)* promoters, respectively, for expression in transgenic *Arabidopsis thaliana* and *Camelina sativa*.

**Results:**

Agrobacterium-mediated transformation successfully generated Arabidopsis and Camelina lines that overexpressed *ZmLEC1* under the control of a seed-specific promoter. This overexpression does not appear to be detrimental to seed vigor under laboratory conditions and did not cause observable abnormal growth phenotypes throughout the life cycle of the plants. Overexpression of *ZmLEC1* increased the oil content in mature seeds by more than 20% in Arabidopsis and 26% in Camelina.

**Conclusion:**

The findings suggested that the maize master regulator, *ZmLEC1,* driven by a downstream seed-specific promoter, can be used to increase oil production in Arabidopsis and Camelina and might be a promising target for increasing oil yield in oilseed crops.0

**Electronic supplementary material:**

The online version of this article (10.1186/s13068-018-1049-4) contains supplementary material, which is available to authorized users.

## Background

Many plants accumulate oils—triacylglycerols (TAGs)—in the seeds, and such seed oils have many important uses. The global production of vegetable oils is more than 180 million tons per year, with soybean, palm seed, and rapeseed oil accounting for almost 80% (http://www.fas.usda.gov/data/ oilseeds-world-markets-and-trade). An increasing fraction of vegetable oils are used for production of biodiesel. Biodiesel is an excellent fuel, but compared to lignocellulosic biofuels, its yield per hectare is still low. To meet the growing demand for vegetable oils both for biodiesel and other uses, there is a need to improve the yield from oil crops.

TAGs are produced in biosynthetic pathways that are generally well understood. A biotechnological approach to increase oil production in seeds is to increase the expression of biosynthetic enzymes that are limiting and represent bottlenecks in the metabolic pathways, and multiple studies have taken that approach [1–4]. However, given that there are many enzymes and cofactors involved and not restricted to only one bottleneck, such an approach is often not the most efficient. An alternative approach is to overexpress transcription factors that control entire pathways. Many biosynthetic pathways are controlled by master regulators, which are transcription factors that control the expressions of other transcription factors. Such master regulators are ideal targets for engineering of plants with increased activity in a desired pathway since ideally only one gene needs to be upregulated to control the expression of multiple genes located downstream of the master regulator encoded by this gene. In addition, by targeting master regulatory transcription factors, a whole pathway can be upregulated even if not all the enzymes, cofactors, transporters, and lower-level transcription factors are known. In TAG biosynthesis, several high-level transcription factors have been identified including wrinkled 1 (WRI1) and leafy cotyledon 1 (LEC1). LEC1 works upstream of WRI1, but both can be considered master regulators for TAG production in seeds. Several groups have tried to overexpress these transcription factors and obtained increased oil production [5–10]. However, in most cases, these studies have made use of strong constitutive promoters, which cause the target genes to be expressed in various tissues and lead to adverse effects on growth or development. Therefore, it is desirable to overexpress the master regulator only in the target tissues or cell types. One approach to tissue-specific expression is to drive the master regulator with a promoter of a downstream-induced gene. In this approach, where we have designated an ‘artificial positive feedback loop,’ the target master regulator will induce its own expression after it is first induced by endogenous transcription factors, and in principle, the master regulator can be expressed to very high levels but only in the cell types where it was expressed in the first place. We have shown that this approach is highly efficient in engineering plants to produce fiber cells with high density of secondary cell walls [11, 12]. We have also shown that the same principle can be used to produce plants that accumulate high amounts of leaf wax on leaf surfaces [13]. In both these cases, the engineering worked significantly better than what had previously been achieved with strong constitutive promoters, which had led to adverse effects on growth. Given the reported experience with the increasing oil production by overexpression of master regulators, we hypothesized that the artificial positive feedback approach could give better results. Indeed, a recent report by van Erp and coworkers used a construct where WRI1 was driven by a promoter from a sucrose synthase gene that is a known downstream-induced gene of WRI1 [14]. The resulting plants had about 10% higher seed oil content than control plants and did not exhibit poor growth and development. We hypothesized that using LEC1 which works upstream of WRI1 and controls more genes required for oil seed development and TAG biosynthesis might give even better results. Overexpression of a maize *LEC1* homolog driven by oleosin (OLE) or early embryo protein (EAP1) promoters has been reported; however, while substantial increases in seed oil were observed, the plants showed poor seed germination and leaf growth [10]. In the present study, we tested the effect of overexpressing a *LEC1* ortholog from maize (*ZmLEC1*) using two different downstream promoters from Arabidopsis (*Arabidopsis thaliana*). We transformed both the model plant Arabidopsis and the oilseed crop Camelina (*Camelina sativa*). In both cases, we achieved more than 20% increased oil content in the seeds, and we did not observe any adverse effects on growth and development. These results highlight the potential application of seed-specific overexpression of *LEC1* for increasing oil production in major crops.

## Results

### Selection of promoters

The ideal promoter for the type of positive feedback we wanted to test should have the following properties: (1) be a direct or indirect target of the endogenous LEC1 and its orthologs, (2) be involved in oil biosynthesis or storage, (3) not be expressed in other tissues than those in developing seeds, (4) be relatively strong. We investigated published data regarding the downstream targets of LEC1 and WRI1 from Arabidopsis (itself a target of LEC1) [15] and identified several candidate genes: *ACP5* (At5g27200, acyl carrier protein 5), *SUS2* (At5g49190, sucrose synthase 2), *PER* (At4g25980, peroxidase superfamily protein), hypothetical protein (At3g63040, LOCATED IN endomembrane system protein), *SCPL17* (At3g12203, serine carboxypeptidase-like 17), *PER1* (At1g48130,1-cysteine peroxiredoxin 1), *BZIP67* (AT3G44460, basic leucine zipper transcription factor 67), *KCS18* (AT4G34520, 3-ketoacyl-CoA synthase 18).

Some of these genes encode proteins that are key enzymes in the fatty acid biosynthetic pathway, which usually coexpress with transcriptional factors regulating fatty acid biosynthesis in seeds. We have used ATTED-II (atted.jp) to analyze coexpression patterns [16]. For example, ACP5 is a key protein directly involved in TAG biosynthesis and it is highly coexpressed with the WRI1 transcription factor (At3g54320) in walking-stick seed and torpedo embryo (http://atted.jp/cgibin/coexpression_viewer.cgi?loc1=832778&loc2=824599). SCPL17 is expressed almost exclusively in siliques in Arabidopsis [17].

**Fig. 1:**
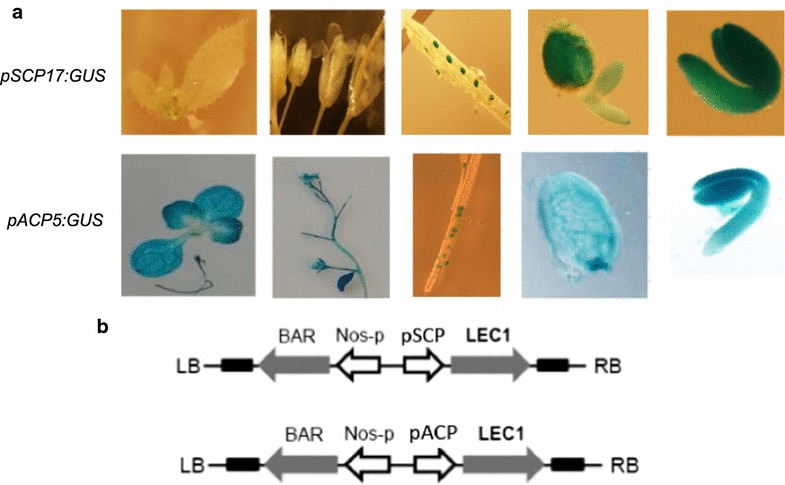
Histochemical analysis of GUS expression under the control of *pSCP17* and *pACP* promoters in leaves, flowers, siliques, endosperm and embryo (**a**), and T-DNA constructs designed for seed-specific overexpression of *ZmLEC1* (**b**). *LB* left border, *BAR* Basta^®^ resistance gene, *pSCP* promoter of *SCP17*, *LEC1* leafy cotyledon 1, *RB* right border

To verify tissue-specific expression of these promoters, we fused them with *GUS* (beta-glucuronidase) gene and transformed Arabidopsis plants. The GUS analysis showed activity with all the chosen promoters, except for that of *KCS18*. Plants transformed with *pSCPL17:GUS* showed a very specific expression restricted only to developing seeds, whereas transformation with *pACP5:GUS* likewise resulted in high expression in developing seeds but also showed some expression in vegetative tissues (Fig. 1a). Since the GUS staining results indicated that *SCPL17* and *ACP5* had the desired properties we selected these promoters to drive the expression of *ZmLEC1* in transgenic Arabidopsis and Camelina plants.

### Generation of transgenic Arabidopsis expressing ZmLEC1

After we had confirmed the expression patterns for the two promoters, they were fused individually to *ZmLEC1* for overexpression in Arabidopsis and Camelina (Fig. 1b). We hypothesized that the validation of nonhost-derived promoters and transcription factors would increase the chance that obtained phenotype could be transferable to a large diversity of plant species while minimizing potential silencing of the transgene and endogenous genes. Likewise, the use of a protein from a distant species could minimize the risk of undesired posttranslational modifications.

**Fig. 2:**
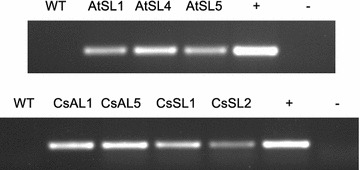
Detection of transgene by PCR analysis of genomic DNA isolated from wild type (WT), transgenic Arabidopsis plants (AtSL1, AtSL4, AtSL5) and transgenic Camelina plants (CsAL1, CsAL5, CsSL1, CsSL2) transformed with *ZmLEC1* constructs. Plasmid positive controls (+) and nontemplate controls (−) were included

The binary vectors containing *ZmLEC1* under the control of a seed-specific *AtSCPL17 (pSCP17)* or *AtACP5 (pACP5)* promoter were introduced into Arabidopsis and Camelina using the Agrobacterium-mediated floral dip method. Transgenic Arabidopsis and Camelina T1 seedlings were selected by hygromycin and Basta, respectively, supplemented in the medium, and resistant lines were confirmed by PCR amplification (Fig. 2).

### *ZmLEC1* expression boosts seeds oil content in Arabidopsis and Camelina seeds

Five to six T1 lines were selected for Arabidopsis and Camelina expressing *pSCPL17:ZmLEC1* and for Camelina expressing *pACP5:ZmLEC1*. Six T2 plants from each of these lines were screened for elevated seed oil content using a nuclear magnetic resonance (NMR) analyzer. For each construct, the transgenic lines exhibited a range in percentage seed oil content. T2 lines with the highest percentage oil content were then taken to produce the T3 generation. Three lines representing transgenic Arabidopsis expressing *pSCPL17:ZmLEC1* (AtSL1, AtSL4, AtSL5) and two lines representing Camelina expressing *pSCP17:ZmLEC1* (CsSL1, CsSL2) or *pACP5:ZmLEC1* (CsAL1, CsAL5) were selected for further studies. Homozygous T2 plants harboring a single insertion were identified for each of the selected lines by segregation analysis.

**Fig. 3:**
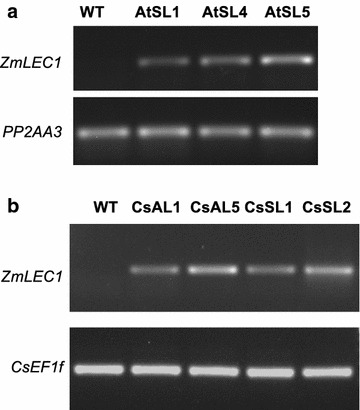
Expression of *ZmLEC1* in developing transgenic Arabidopsis (**a**) and Camelina (**b**) seeds. **a** Total RNA was isolated from developing seeds 6–9 DAF and subjected to RT-PCR analyses. The *PP2AA3* gene was used as a housekeeping gene to confirm the quality and quantity of RNA. **b** Total RNA was isolated from developing seeds 15 DAF. The *CsEF1f* gene was used as a housekeeping gene to confirm the quality and quantity of RNAs. WT is the wild type, AtSL are independent homozygous T3 lines expressing the *pSCPL17:ZmLEC1* transgenes in Arabidopsis. CsAL and CsSL are independent homozygous T3 lines expressing the *pSCPL17:ZmLEC1* and *pACP5:ZmLEC1* transgenes, respectively, in Camelina

To confirm the expression of the target gene (*ZmLEC1*), reverse transcription-PCR (RT-PCR) analysis was performed on developing Arabidopsis siliques containing seeds in developmental stages 6–9 [18] and Camelina developing seeds at 15 days after flowering (DAFs) (Fig. 3). *ZmLEC1* transcripts were detected in all the selected lines and absent in the wild type.

**Fig. 4:**
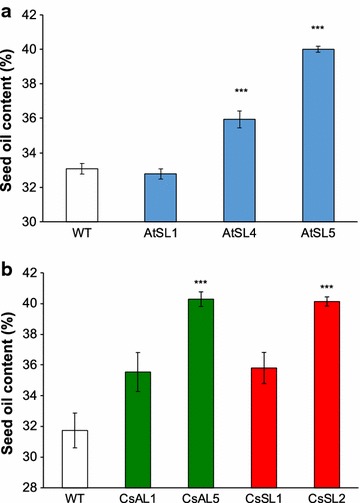
Seed oil content of independent transgenic lines expressing *ZmLEC1* in Arabidopsis (**a**) and Camelina (**b**). Oil content in mature seeds of wild-type plants and transformants was determined by NMR. Wild-type and transgenic plants are designated as explained in legend to Fig. 3. Significant differences compared to wild type (ANOVA and Dunnett’s test) are indicated: ***P < 0.001. Error bars indicate standard error [*n* = 6 except for Camelina wild type (*n* = 30)]

Six individual Arabidopsis T3 plants of each of the *pSCPL17:ZmLEC1* transformed lines and six wild-type Arabidopsis plants, and six individual Camelina T3 plants of each of the *pACP5:ZmLEC1* and the *pSCP17:ZmLEC1* transformed lines and 30 wild-type Camelina plants were grown under controlled conditions. Individual plants were arranged randomly in the trays to avoid edge effects that could bias the results. The selected AtSL4 and AtSL5 lines exhibited statistically significant (*P* < 0.001) increases in percentage seed oil content compared to the wild type (Fig. 4a). The seed oil content in AtSL5 reached 40%, which is an approximately 20% increase over the wild type (95% confidence interval 18–24%). The selected CsAL5 and CsSL2 lines also exhibited statistically significant (*P* < 0.001) increases in percentage seed oil content compared to the wild type. The seed oil content in CsAL5 and CsSL2 was more than 40%, which is an approximately 26% increase over the content found in wild-type seeds (95% confidence interval 18–37%) (Fig. 4b).

### Overexpressing *ZmLEC1* does not adversely affect seed vigor or plant growth

**Fig. 5:**
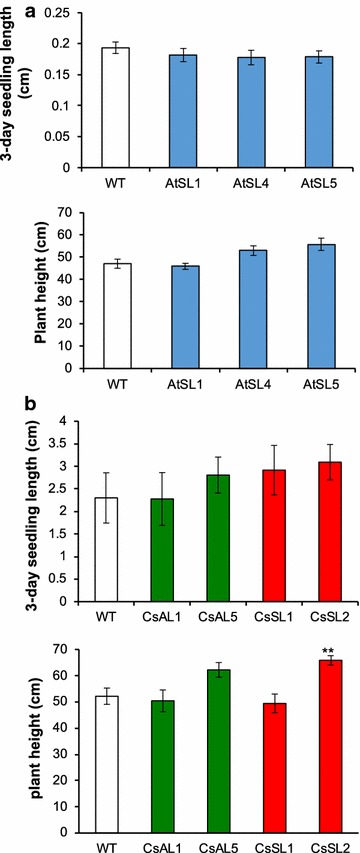
Effect of *ZmLEC1* expression on early seedling growth rate and plant height at maturity in Arabidopsis (**a**) and Camelina (**b**). Wild-type and transgenic plants are designated as explained in legend to Fig. 3. Significant differences compared to wild type (ANOVA and Dunnett’s test) are indicated: **P < 0.01. Error bars indicate standard error [*n* = 6 except for Camelina wild type (*n* = 30)]

Considering the adverse growth effects reported in previous studies of overexpression of WRI1 and LEC1 [10, 19], it was important to assess the phenotype during the whole life cycle of the transgenic plants. We did not observe any obvious phenotypical differences in the transgenic plants (Additional files 1, 2, 3) and we specifically measured seedling and stem growth. Nearly all seeds germinated, and seedling length 3 days postgermination showed no significant difference between the transgenic plants and the control plants (Fig. 5a). The transgenic plants showed a tendency to be taller than control plants, but this was only significant in one of the Camelina lines (Fig. 5b).

### Overexpressing *ZmLEC1* plants provide increased oil yield

**Fig. 6:**
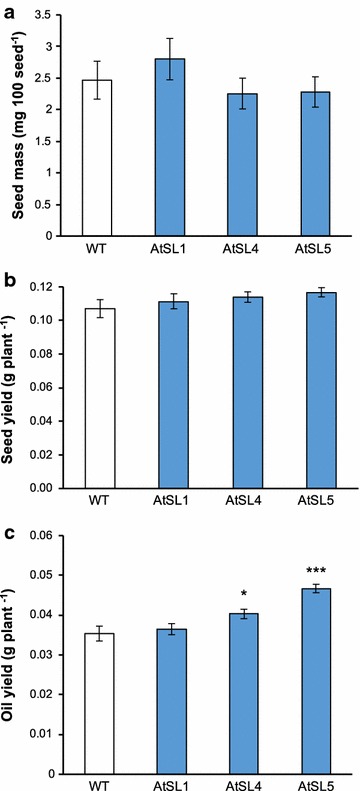
Effects of *ZmLEC1* expression on average seed mass per 100 seeds (**a**), seed yield per plant (**b**) and oil yield per plant (**c**) in Arabidopsis. Wild-type and transgenic plants are designated as explained in legend to Fig. 3. Significant differences compared to wild type (ANOVA and Dunnett’s test) are indicated: *P < 0.05, ***P < 0.001. Error bars indicate standard error (*n* = 6)

**Fig. 7:**
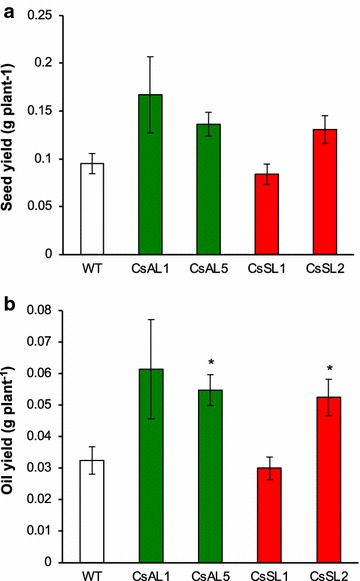
Effects of *ZmLEC1* expression on average seed yield per plant (**a**) and oil yield per plant (**b**) in Camelina. Wild-type and transgenic plants are designated as explained in legend to Fig. 3. Significant differences compared to wild type (ANOVA and Dunnett’s test) are indicated: *P < 0.05. Error bars indicate standard error [*n* = 6 except for wild type (*n* = 30)]

We examined additional traits of the transgenic lines to evaluate the total oil yield on a per plant basis as it depends on seed number per plant, seed size and oil fraction per seed. Analysis of 100-seed-weight showed that the expression of *ZmLEC1*, using downstream promoters, does not result in a significant change in the average seed mass of Arabidopsis (Fig. 6a). In addition, all the seeds produced by each plant were collected to determine total seed yield in grams per plant (Figs. 6b and 7a). There was a trend towards larger total seed yield in both species, but none of the differences were significant. From the total seed yield per plant (gram) and the seed oil content (percentage of seed weight), we calculated the total oil yield in grams per plant (Figs. 6c and 7b). The data show that the total oil yield per plant is significantly (*P* < 0.05) increased by 13 and 32% in the AtSL4 and AtSL5 lines, respectively (Fig. 6c). In the Camelina CsAL5 and CsSL2 lines, the total oil yield per plant was also significantly (*P* < 0.05) increased (Fig. 7b). Because of variations in both seed yield and oil content, the relative increase in seed yield per plant cannot be calculated with high precision. It will be important to repeat these studies in Camelina plants grown under field conditions.

### *ZmLEC1* overexpression upregulates the downstream oil-related genes in developing seeds

**Fig. 8:**
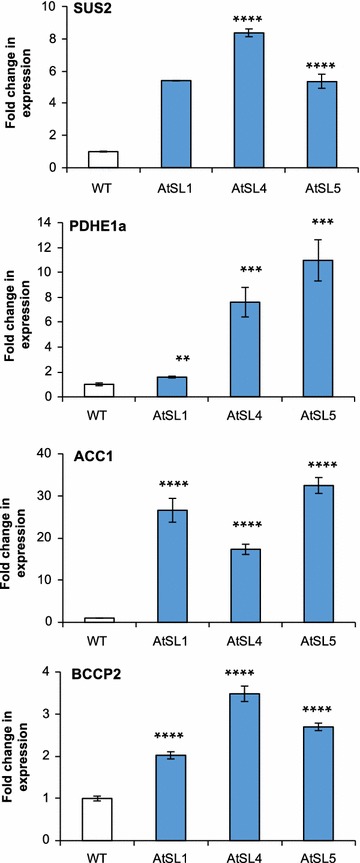
Analysis of SUS2, PDH E1α, BCCP2, and ACC1 expression using quantitative reverse transcription-PCR on developing siliques of Arabidopsis. Wild-type and transgenic plants are designated as explained in legend to Fig. 3. Significant differences compared to wild type (Student’s *t* test) are indicated: **P < 0.01, ***P < 0.001 and ****P < 0.0001. Error bars indicate standard deviation, *n* = 6

**Fig. 9:**
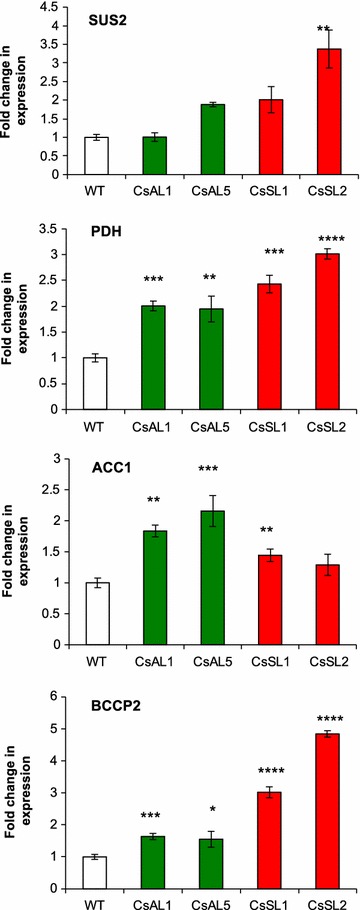
Analysis of SUS2, PDH E1α, BCCP2, and ACC1 expression using quantitative reverse transcription-PCR on developing seeds of Camelina transgenic lines. Wild-type and transgenic plants are designated as explained in legend to Fig. 3. Significant differences compared to wild type (Student’s *t* test) are indicated: **P < 0.01, ***P < 0.001 and ****P < 0.0001. Error bars indicate standard deviation, *n* = 6

The transcript levels of a selection of oil-related genes were measured to examine the impact of the *ZmLEC1* overexpression on these genes. In the transgenic Arabidopsis lines, several downstream genes regulated by *AtLEC1* were upregulated by about two to tenfold compared with the wild-type plants: the sucrose synthase gene *AtSUS2* (At5g49190), plastidic pyruvate dehydrogenase (*PDH*) E1a subunit (At1g01090) involved in late glycolysis, and acetyl CoA carboxylase (ACCase) *BCCP2* subunit (At5g15530) involved in de novo fatty acid synthesis. The chain elongation related gene, *ACC1* (At1g36160), was upregulated 20- to 30-fold (Fig. 8). In the transgenic Camelina lines, the corresponding *CsSUS2* (XM_010441988), *CsPDH* (XM_010458872), *CsACC1* (XM_010501769), and *CsBCCP2* (XM_010455390) genes were all upregulated by about two to fivefold compared to those in wild-type plants (Fig. 9). In general, expression levels of the analyzed genes did not change as dramatically in Camelina as in Arabidopsis even though the increase in oil content was similar in the two species.

Experimental manipulation of ZmLEC1 caused transcriptional changes of genes coding for enzymes participating in sucrose metabolism, glycolysis, and FA biosynthesis, suggesting an enhanced carbon flux towards FA biosynthesis in tissues overexpressing ZmLEC1.

## Discussion

Many scientists have recognized that overexpressions of transcription factors provide an attractive solution for increasing plant oil production compared to the overexpressions of pathway enzymes [14, 20, 21]. However, promoters must be chosen carefully to avoid negative effects while obtaining sufficient expression levels in target tissues. Van Erp and coworkers used a construct with a sucrose synthase 2 (Sus2) promoter to drive Arabidopsis *WRI1* [14]. They obtained an approximately 10% increase in oil content in Arabidopsis seeds with this promoter–gene combination. An and Suh used the promoter of SiW6P (encoding linoleic acid desaturase) from *Sesamum indicum* to drive the expression of the Arabidopsis *WRI1* in Camelina [22]. They obtained seeds from 13 different transgenic lines, and the best had a 10.1% increase in seed fatty acid esters. A patent application described a variety of promoters used to drive WRI1 homologs from corn and other plants in Arabidopsis leading to 4.9% increase in oil with *pSUC2:ZmODP* (ODP is a WRI1 homolog) [23]. They also described the construct *pSCP1:ZmODP*, but oil was only increased by up to 2.6% in the best line. In our study, we tested several seed-specific promoters and used them to drive the expressions of *ZmWRI1*, *AtWRI1*, and *ZmLEC1* in Arabidopsis and Camelina. The most promising preliminary results were obtained with ZmLEC1, which works upstream of WRI1. To identify the best promoters, we looked at the expression pattern using promoter-GUS constructs. Based on the GUS analysis and comparison of different lines, we could identify pSCPL17 (serine carboxypeptidase-like 17, At3g12203) as a strong and very seed-specific promoter. Another good promoter based on our analysis was pACP5 (acyl carrier protein 5, At5g27200), which is stronger but less seed specific. These promoter combinations with *ZmLEC1* worked better than the previously published designs described above. In Arabidopsis lines transformed with *pSCPL17:ZmLEC1*, we find an increase in oil content of 21%, compared to a maximum of 10% in the cited study [14]. In Camelina, a 26% increase in seed oil content was obtained with both *pACP5:ZmLEC1* and *pSCPL17:ZmLEC1* designs, compared to the 10.1% obtained in the cited study [22]. With our transgene designs, better results were obtained with ZmLEC1, compared to those obtained with AtWRI1 or ZmWRI1, presumably because it works further upstream. Shen and coworkers obtained up to 30.6% increase in seed oil content in maize overexpressing *WRI1* with seed-specific oleosin promoter and did not observe negative effects on growth or yield [10]. This interesting result is difficult to compare with results obtained with Arabidopsis and Camelina where seed oil content is naturally an order of magnitude higher than that observed in maize seeds, and where such high oil yield increases have not previously been reported. Obviously, it will be interesting to test our promoter–transcription factor combinations in other crops such as canola, maize, and soybean.

In general, it is important to choose a promoter of the appropriate strength and specificity for constructs such as those described here. The ZmLEC1 overexpression can be combined with other engineering constructs, in particular, the overexpression of diacylglycerol acyl transferase (DGAT1) and downregulation of the lipase Sugar-dependent 1 (SDP1) that were shown to increase the accumulation of oil when combined with overexpression of AtWRI1 [14].

## Conclusions

Arabidopsis and Camelina lines overexpressing *ZmLEC1* under the control of an Arabidopsis seed-specific promoter increased oil content in mature seeds by more than 20% in Arabidopsis and 26% in Camelina. Overexpression of *ZmLEC1* does not appear to be detrimental to seed vigor under laboratory conditions. Furthermore, no abnormal growth phenotypes were observed throughout the life cycle of the plants. The findings suggested that a master regulator, *ZmLEC1,* driven by a downstream seed-specific promoter, can be used to influence oil production in Arabidopsis and Camelina and might be a promising transgene design for increasing oil production in various crops.

## Methods

### Plant material and growth conditions

Wild-type (Col-0) Arabidopsis [*Arabidopsis thaliana* (L.) Heynh.] seeds were surface sterilized, sowed on agar plates containing one-half-strength Murashige and Skoog salts (Sigma-Aldrich) containing 1% sucrose, and incubated in darkness for 3 days at 4 °C. The plates were then placed in a growth chamber set to 16-h/8-h day/night cycle, photosynthetic photon flux density of 250 µmol m^−2^ s^−1^, and 70% relative humidity. After 12 days, each seedling was transplanted to 7-cm^2^ pots. Individual plants in each pot were arranged randomly in a tray. When plants began to flower, a 60-cm stick was inserted in each pot to tie the stems, and at maturity, the pot was placed inside an upright rectangular transparent, perforated glassine bag (60 × 6 cm). The bag was sealed around the lower stem prior to seed shedding to ensure that all the seeds from each plant were retained.

Wild-type Camelina [*Camelina sativa* (L.) Crantz, cultivar ‘Suneson’] seeds were surface sterilized, sowed on agar plates containing one-half-strength Murashige and Skoog salts (Sigma-Aldrich) supplemented with 1% sucrose, and incubated in darkness for 3 d at 4 °C. The plates were then placed in a growth chamber set to 16-h/8-h day/night cycle, photosynthetic photon flux density of 250 µmol m^−2^ s^−1^, and 70% relative humidity. After 5 days, each seedling was transplanted to 15-cm^2^ pots. Individual plants in each pot were arranged randomly in a tray.

### Creation of DNA constructs and plant transformation

A 1.9 kb promoter for *AtSCPL17* (At3g12203; pSCP) and a 2.5 kb promoter for *AtACP5* (At5g27200; pACP) were amplified by PCR from Arabidopsis genomic DNA using the SCP-F/R and ACP5-F/R primer pairs, respectively (Additional file 4), and cloned into pCR_Blunt (Thermo Fisher Scientific). The *ZmLEC1*-coding sequence was synthesized directly based on the amino acid sequence from maize (*Zea mays* leafy cotyledon 1, AF410176) without stop codon. The sequences corresponding to those of attb1 and attb2 were inserted during synthesis at the 5′-end and 3′-end of the *ZmLEC1*-coding sequence, respectively. The synthesized *ZmLEC1* was then subcloned into the Gateway pDONR221-P1P2 (Zeocin) entry vector by BP recombination (Life Technologies).

Two versions of vectors were generated for hygromycin and Basta selection of transformed Arabidopsis and Camelina lines, respectively. For the spectinomycin selection of bacteria, a spectinomycin marker was inserted in the backbone of the pA6-pC4H::GW vector [24] at the unique SacII restriction site. The spectinomycin marker was amplified from the pTKan vector [11] using the primer pair F-Spect-pA6-SacII/R-Spect-pA6-SacII and inserted at the SacII restriction site using Gibson assembly method (New England Biolabs) to generate pA6Spect-pC4H::GW vector. The resulting vector was then digested by HindIII and AvrII to replace the pC4H promoter by p*SCP* and p*ACP* to generate two new vectors: pA6Spect-pSCP::GW and pA6Spect-pACP::GW. The Basta-resistant vectors were generated by the replacement of the hygromycin marker in pA6Spect-pSCP::GW and pA6Spect-pACP::GW by that of BastaR. Both destination vectors were digested by *Apa*I and *Ase*I to remove the hygromycin selection cassette. The BASTA marker was amplified by PCR using the primer pair F-Basta-pA6-ApaI/R-Basta-pA6-AseI and pEARLYGATE [25] as template, then inserted between the ApaI and AseI to generate two destination vectors: pA6Spect-pSCP::GW-BASTA and pA6Spect-pACP::GW-BASTA.

The pA6Spect-pSCP::GUS and pA6Spect-pACP::GUS vectors were generated by LR cloning (Life Technologies) using pA6Spect-pSCP::GW and pA6Spect-pACP::GW destination vectors and a pDONR221-L1GUSL2 entry vector. Overexpression constructs containing the coding sequence of *ZmLEC1* fused to the Arabidopsis promoters were created the same way by LR cloning using pA6Spect-pSCP::GW, pA6Spect-pSCP::GW-BASTA, pA6Spect-pACP::GW, and pA6Spect-pACP::GW-BASTA destination vectors, and the synthesized *ZmLEC1* entry clone.

The recombination construct pA6Spect-XXX and pA6Spect-XXX-BASTA vectors were transformed into *Agrobacterium tumefaciens* strain GV3101 by heat shock and into Arabidopsis and Camelina, respectively, by the floral dip method [26]. Arabidopsis and Camelina transformants were selected on 1/2 MS medium supplemented with hygromycin (30 µg/mL) or Basta^®^ (glufosinate ammonium; 30 µg/mL), respectively.

### Histochemical GUS assay

For histochemical GUS staining, fresh samples from various tissues, including siliques, were incubated in X-Gluc solution [1 mM 5-bromo-4-chloro-3-indolyl-β-d-glucuronic acid, 50 mM phosphate buffer (pH 7.0), 2 mM potassium ferricyanide, 2 mM potassium ferrocyanide, 0.2% (v/v) Triton X-100] at 37 °C overnight. The staining buffer was then carefully removed, and the samples were washed three times (2-h washes) with ethanol:acetic acid (7:3) and stored in 95% ethanol.

### Genotyping

DNA extraction was conducted according to the manual of REDExtract-N-Amp™ Plant PCR Kits (Sigma-Aldrich), and PCR carried out with leaf disk extract as template and *ZmLEC1* F/R primer pairs. The PCR condition was 30 cycles of denaturation at 94 °C for 30 s, annealing at 55 °C for 30 s, and elongation at 72 °C for 30 s.

### Transcript analysis

DNase-treated total RNA was extracted from Arabidopsis siliques at 6 DAF, which corresponds to developmental stages 6–9 [18] and Camelina developing seeds at 15 DAF using RNeasy kit from Qiagen. The synthesis of single-stranded complementary DNA was carried out using iScript Reverse Transcription Supermix for RT-qPCR from BIO-RAD. Primers were designed using the IDT DNA Real-Time PCR primer design tool (http://www.idtdna.com/scitools/Applications/RealTimePCR) (Additional file 4).

Quantitative real-time PCRs were performed on a STEPONE CFX96 Real-Time system (Applied Biosystems) and QuantiFast SYBR Green PCR kit from Qiagen following the manufacturers recommendation.

### Seed oil content analysis

The percentage of oil in seeds was determined with a mini-spec mq10 nuclear magnetic resonance (NMR) analyzer (Bruker Optics Inc., Houston, TX, USA). The mini-spec was calibrated by linear regression of NMR signals to weighed samples of pure Camelina oil following a general protocol provided by Bruker. Each seed sample was weighed and placed in the NMR tube and then measured against the calibration curve to determine the oil content. Three seed samples were analyzed from each plant, with the number (*n*) of plants given in the figure legends. Calibration standards and seed samples were tempered at 40 °C for 0.5–1 h before NMR measurement. The mini-spec was operated at a resonance frequency of 9.95 MHz and was maintained at 40 °C. Each measurement takes about 15 s.

### Seed germination and seedling growth assays

The surface-sterilized seeds were plated on agar plates containing one-half-strength Murashige and Skoog salts (Sigma-Aldrich) containing 1% sucrose, and incubated in darkness for 3 days at 4 °C. The plates were then placed vertically in a growth chamber set to 20 °C with constant low light (photosynthetic photon flux density of 10 µmol m^−2^ s^−1^) for 3 days. Then the seedling length was measured.

### Statistical analyses

The number of replicates (*n*) and the standard errors are shown for most measurements. One-way ANOVA was used to assess the differences between genotypes for measurements of seed percentage oil content, 3-day seedling length, plant height, seed mass, seed yield, oil yield and fold-change in gene expression. When the ANOVA showed significant differences (*P* < 0.05) the individual lines were compared to wild type by Dunnett’s test or Student’s *t* test as indicated in the figure legends, with significance levels below 0.05 indicated in the figures. ANOVA and multiple comparisons were done using XLSTAT (Addinsoft, New York). Confidence intervals of ratios were calculated with GraphPad using Fieller’s Method.

## Additional files

**Additional file 1.** Phenotypes of mature Arabidopsis plants.

**Additional file 2.** Phenotypes of 45-days-old Camelina plants.

**Additional file 3.** Phenotypes of mature Camelina plants.

**Additional file 4.** Primers used for cloning and qPCR.
