# Supplementary material for: A transgene design for enhancing oil content in Arabidopsis and Camelina seeds
Source: Biotechnol Biofuels. 2018 Feb 21;11:46. doi: 10.1186/s13068-018-1049-4 (PMC5820799; doi:10.1186/s13068-018-1049-4)
Supplement: Supplementary file 1 — Additional file 1. Phenotypes of mature Arabidopsis plants. [file 13068_2018_1049_MOESM1_ESM.pdf]

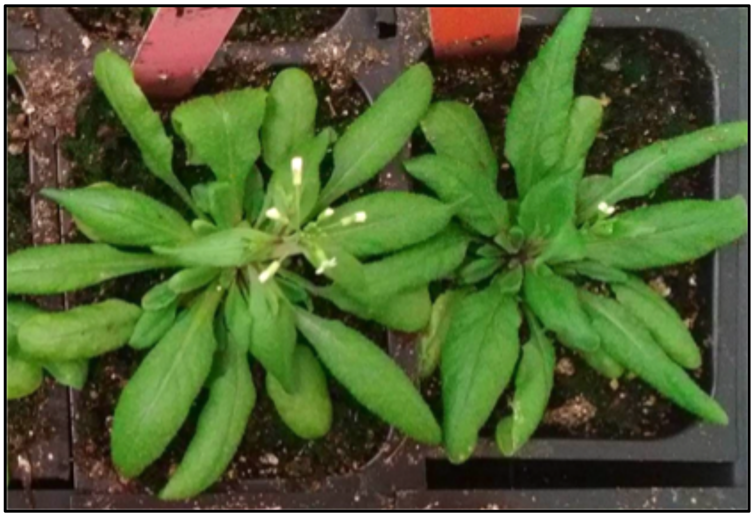

ATSL5 Wild type

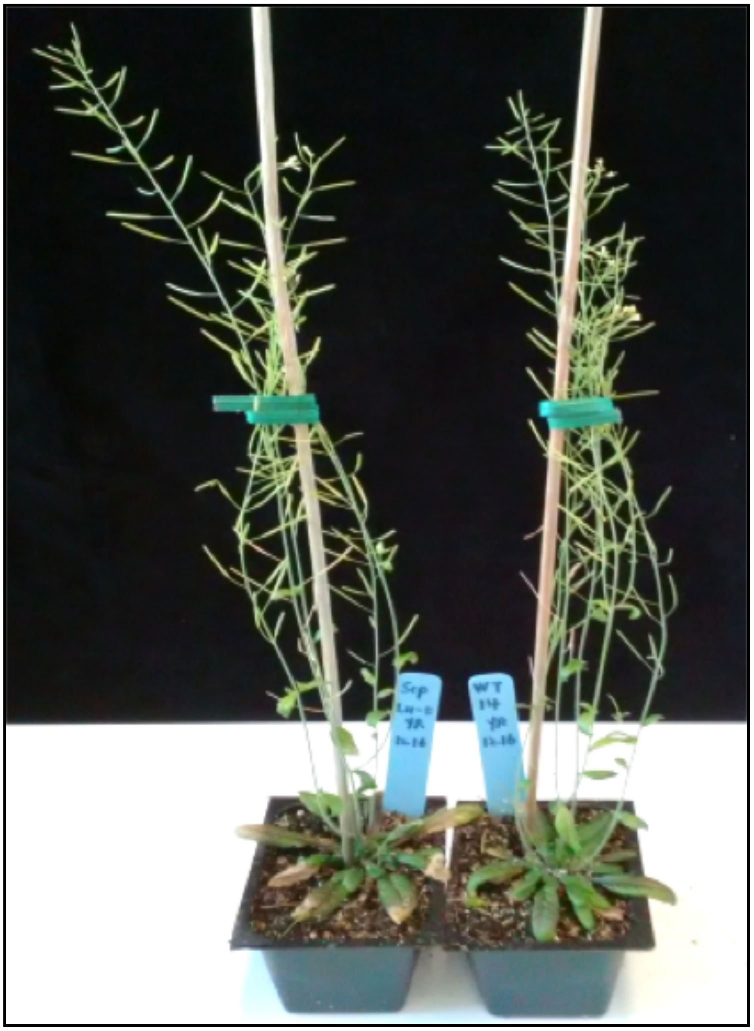

ATSL4 Wild type

Additional File 1: Phenotypes of arabidopsis *pSCP17:ZmLEC1* and Col-0 wild type. The plants in rosette stage are T2 generation and the mature plants are T3 generation.
