# Supplementary material for: A transgene design for enhancing oil content in Arabidopsis and Camelina seeds
Source: Biotechnol Biofuels. 2018 Feb 21;11:46. doi: 10.1186/s13068-018-1049-4 (PMC5820799; doi:10.1186/s13068-018-1049-4)
Supplement: Supplementary file 2 — Additional file 2. Phenotypes of 45-days-old Camelina plants. [file 13068_2018_1049_MOESM2_ESM.pdf]

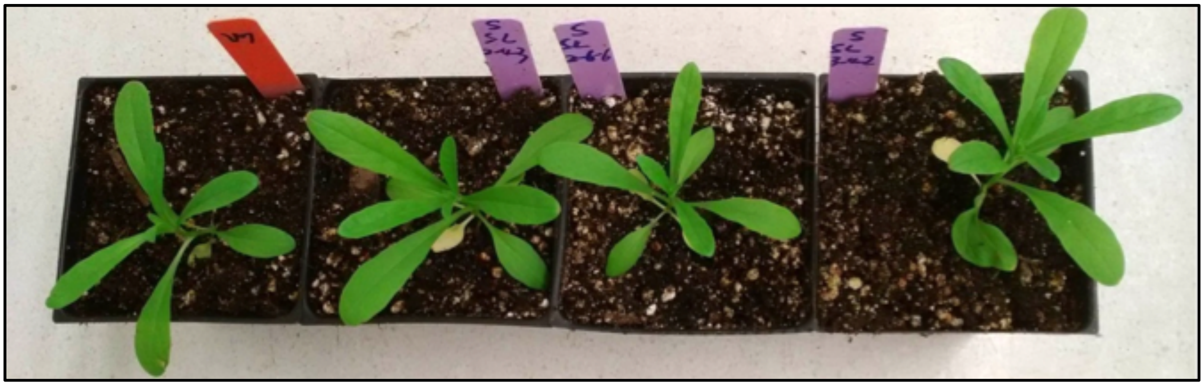

WT CsSL2 CsSL2 CsSL1

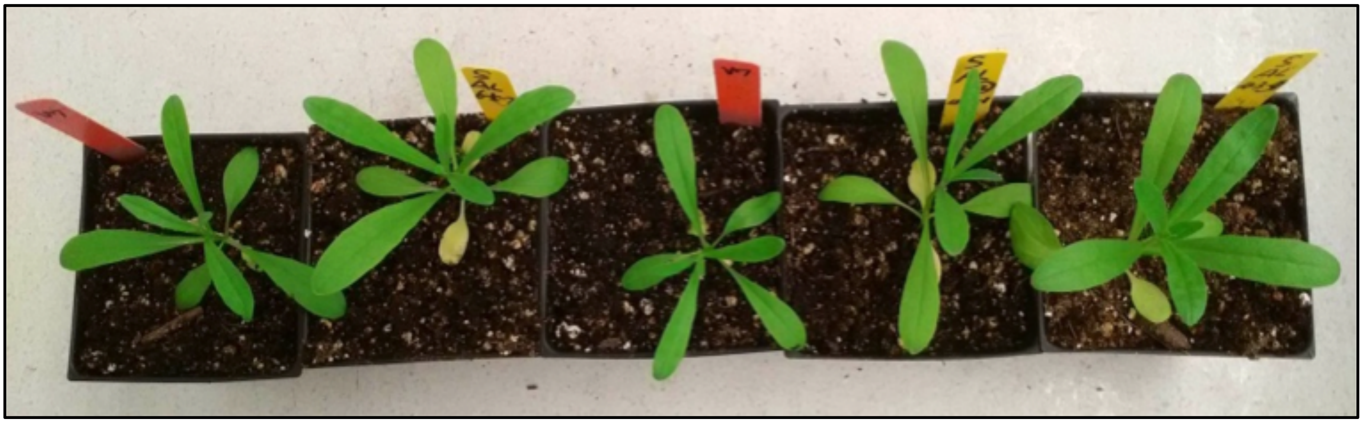

WT CsAL6 WT CsAL1 CsAL4

**Additional File 2: Phenotypes of 45-days-old T3 and wild type (WT) Camelina plants.**
