# Supplementary material for: A transgene design for enhancing oil content in Arabidopsis and Camelina seeds
Source: Biotechnol Biofuels. 2018 Feb 21;11:46. doi: 10.1186/s13068-018-1049-4 (PMC5820799; doi:10.1186/s13068-018-1049-4)
Supplement: Supplementary file 3 — Additional file 3. Phenotypes of mature Camelina plants. [file 13068_2018_1049_MOESM3_ESM.pdf]

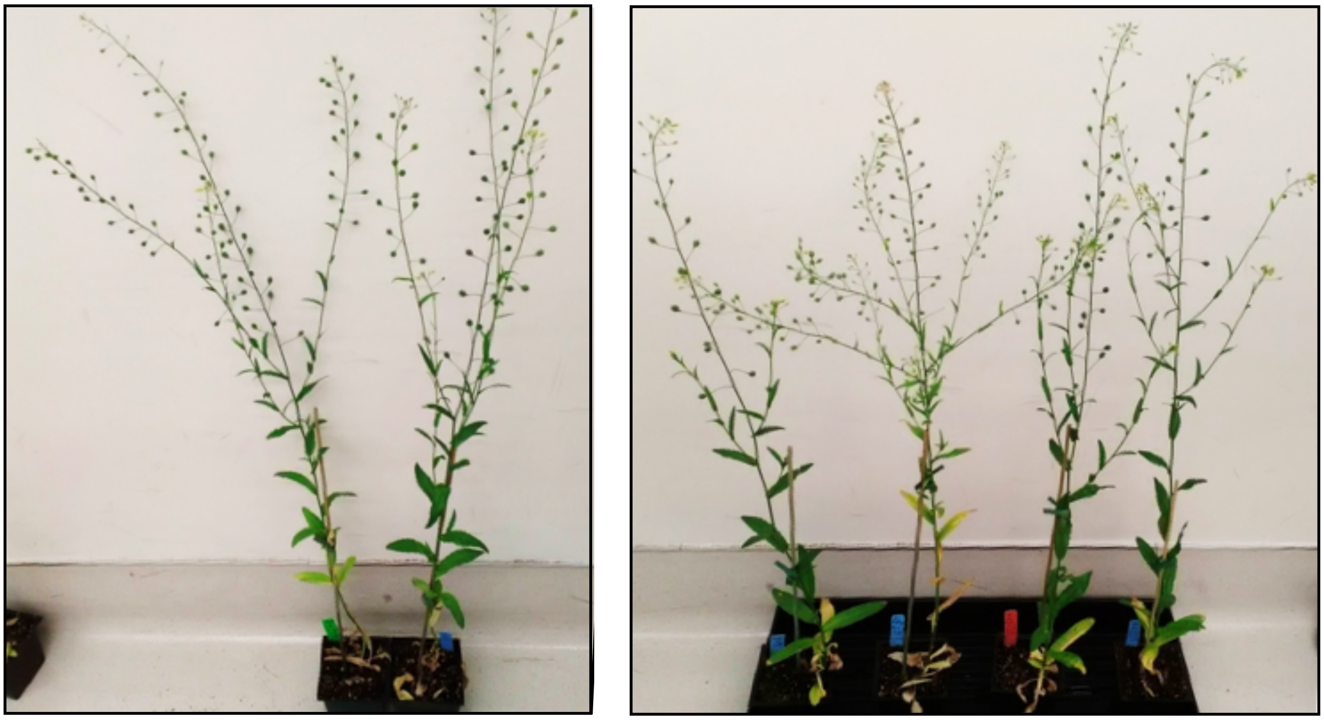

CsAL3 WT

WT CsAL3 CsSL2 CsSL5

Additional File 3: Phenotypes of mature T3 and wild type (WT) Camelina plants.
