# Supplementary material for: A transgene design for enhancing oil content in Arabidopsis and Camelina seeds
Source: Biotechnol Biofuels. 2018 Feb 21;11:46. doi: 10.1186/s13068-018-1049-4 (PMC5820799; doi:10.1186/s13068-018-1049-4)
Supplement: Supplementary file 4 — Additional file 4. Primers used for cloning and qPCR. [file 13068_2018_1049_MOESM4_ESM.pdf]

Additional File 4

Details of primers used.

Primer name	Gene name	Primer sequence (5'-3')
Primer sequences for cloning		
pSCP F	*SCP17*	CCAAGCTTGGAAGAGCTCTTCTCTGGCTGTG
pSCP R		GACCTAGGTCTCTTTGATCAAAAGTTTTT
pACP F	*ACP5*	CCAAGCTTGCATACTCTCTCGTGAACTC
pACP R		GACCTAGGTATCGATCTGATCGAGAG
ZmLEC1 F	*LEC1*	GATGCCAAAGGGAGCAGAGA
ZmLEC1 R		CCCCTTGCATCACCCTCAAA
F-Spect-pA6-SacII	*SpecR*	ccgattttgaaaccgcggCATGATATATCTCCCAATTTGTG
RSpect-pA6-SacII	*SpecR*	ctgcctgtgatcaccgcggTAAGCCTCGTTCGGTTCGT
F-Basta-pA6-ApaI	*BastaR*	ctcggtaccaagcttggggcgcgccGATACATGAGAATTAAG GGAGTC
R-Basta-pA6-AseI	*BastaR*	ctgaattaacgccgaattaatGAGCTTGCATGCCGGTCGATC
Housekeeping gene primers in Arabidopsis		
SAND F	*SAND*	AACTCTATGCAGCATTTGATCCACT
SAND R		TGATTGCATATCTTTATCGCCATC
PP2AA3 F	*PP2AA3*	TAACGTGGCCAAAATGATGC
PP2AA3 R		GTTCTCCACAACCGCTTGGT
ER332		GAGCTGAAGTGGCTTCCATGAC
ER333		GGTCCGACATACCCATGATCC
Primers for semi-quantitative / quantitative PCR of Arabidospsis		
AtACC1 F	*AtACC1*	AGTGAGAATGCATAGGTTGGG
AtACC1 R		CTCGGTATATGTGGACAGTGC
AtBCCP2 F	*AtBCCP2*	GACCCGGTGAACCCCCT
AtBCCP2 R		GTCAACGCTGACTGGTTTTCCAT
AtPDHE1C F	*AtPDHE1C*	ATGTGTGCTCAAATGTATTACCGAGGC
AtPDHE1T R		ACCTTTGCTGAGGGCATGG
AtSUS2 F	*AtSUS2*	GCGGGAAGCAAGAACAATG
AtSUS2 R		GAACAACTCGGTAAAGACCAGGC

Housekeeping gene primers in Camelina		
CsActin F	*CsActin*	ACA ATT TCC CGC TCT GCT GTT GTG
CsActin R		AGG GTT TCT CTC TTC CAC ATG CCA
CsTubulin F	*CsTubulin*	GGGCTAAGGGACATTACACTG
CsTubulin R		GTGTTCCCATACCAGATCCAG
CsEF1α F	*CsEF1α*	GGTAAGGAGATTGAGAAGGAGC
CsEF1α R		CACAGCAAAACGTCCCAATG
Primers for semi-quantitative / quantitative PCR of Camelina		
CsACC1 F	*CsACC1*	CTAAGCCCTGAAGACTACGAAC
CsACC1 R		ATTACCCACCTTGTTTCCCC
CsBCCP2 F	*CsBCCP2*	ACACAGTGGCATCTCCTTTC
CsBCCP2 R		GGTTTTGGCTTTTCCGTTCAG
CsPDHE1T F	*CsPDHE1T*	TGGAGAATAACTTGTGGGCG
CsPDHE1A R		ACCTTCAACACATCCATACCG
CsSUS2 F	*CsSUS2*	CGTTTGCTACTTGTCATGGTG
CsSUS2 R		TTACCCAGTGATTCGGATTGG
